# Future acceptance of automated insulin delivery systems in youths with type 1 diabetes: validation of the Italian artificial pancreas-acceptance measure

**DOI:** 10.1007/s00592-024-02327-9

**Published:** 2024-08-10

**Authors:** Roberto Franceschi, Riccardo Pertile, Marco Marigliano, Enza Mozzillo, Claudio Maffeis, Silvana Zaffani, Carlotta Dusini, Annalisa Antonelli, Francesca Di Candia, Giulio Maltoni, Erika Cantarelli, Nicola Minuto, Marta Bassi, Ivana Rabbone, Silvia Savastio, Stefano Passanisi, Fortunato Lombardo, Valentino Cherubini, Maria Alessandra Saltarelli, Stefano Tumini

**Affiliations:** 1 Department of Pediatrics, S.Chiara Hospital of Trento, APSS, Trentino-Alto Adige, Trento Italy; 2 Clinical and Evaluative Epidemiology Unit, Health Management, APSS, Trento, Italy; 3 Department of Surgery, Dentistry, Pediatrics, and Gynecology, Section of Pediatric Diabetes and Metabolism, University of Verona, Piazzale Aristide Stefani 1, Verona, 37126 Italy; 4 Department of Translational Medical Science, Section of Pediatrics, Università degli Studi di Napoli Federico II, Naples, Italy; 5 Pediatric Unit, IRCCS Azienda Ospedaliero-Universitaria di Bologna, Bologna, Italy; 6 Pediatric Clinic, Department of Neuroscience Rehabilitation Ophthalmology Genetics, Maternal and Child Health, IRCCS Giannina Gaslini, University of Genoa, Genova, Italy; 7 Division of Pediatrics, Department of Health Sciences, University of Piemonte Orientale, Novara, Italy; 8 Department of Human Pathology of Adulthood and Childhood G. Barresi, University of Messina, Messina, Italy; 9 Department of Women’s and Children’s Health, Azienda Ospedaliero-Universitaria, Ospedali Riuniti di Ancona, “G. Salesi Hospital”, Ancona, Italy; 10 Department of Maternal and Child Health, UOSD Regional Center of Pediatric Diabetology, “SS Annunziata” Hospital, Chieti, Italy

**Keywords:** Child, Adolescents, Artificial pancreas, AID, Insulin pump, CSII, Acceptance, Technology

## Abstract

**Aim:**

The purpose of this study was to develop a questionnaire to examine the future acceptance of Automatic insulin delivery systems (AIDs), their perceived usefulness, ease of use, and trust in the device in subjects with type 1 diabetes (T1D).

**Methods:**

A questionnaire in Italian, based on the Technology Acceptance Model, was developed to examine intention to use AIDs, considered as a measure of future acceptance, and its determinants to use the system. A total of 43 questions for children and 46 for parents were included, and a 5-point Likert scale was used.

**Results:**

239 subjects with T1D using multiple daily injections (MDI) or sensor-augmented pump (SAP) and their parents completed the questionnaire. The completion rate was excellent, with almost 100% of items answered. The overall Cronbach’s coefficient for children and adolescents was 0.92 and 0.93 for parents, indicating excellent internal consistency in both groups. Parent-youth agreement was 0.699 (95% confidence interval: 0.689–0.709), indicating a good agreement between the two evaluations. Factor analysis identified measurement factors for the “artificial pancreas (AP)-acceptance labeled benefits and hassles of AIDs, and the internal consistency of the total scale was alpha = 0.94 for subjects with T1D and 0.95 for parents. The level of AP acceptance was more than neutral: 3.91 ± 0.47 and 3.99 ± 0.43 (*p* = 0.07) for youths and parents, respectively (possible score range 1 to 5, neutral score is 3.0). Parents reported higher scores in the benefit items than children-adolescents (*p* = 0.04).

**Conclusions:**

We developed a new questionnaire based on the items available in the literature, and we demonstrated that the “AP-acceptance” reveals a meaningful factor structure, good internal reliability, and agreement between parent–young people evaluations. This measure could be a valuable resource for clinicians and researchers to assess AP acceptance in pediatric patients with T1D and their parents. This patient profiling approach could help to enroll candidates for AIDs with proper expectations and who most likely will benefit from the system.

**Supplementary Information:**

The online version contains supplementary material available at 10.1007/s00592-024-02327-9.

## Introduction

Automated insulin delivery systems (AIDs) allow youths with type 1 diabetes (T1D) to achieve optimal glucose control [1]. Still, barriers related to continuous subcutaneous insulin infusion (CSII), such as catheter insertion-related issues, altered body shape, social acceptance [2], and to continuous glucose monitoring (CGM) as skin irritations, inaccurate readings, and excessive alarms [3–5], can lead to drop-out the AIDs [6]. Perceived benefits and burdens may predict user satisfaction and the sustained use of the novel technology [6]. Exploring the attitudes and feelings of youths with T1D and those of their caregivers towards AIDs, could help to individuate who is ready to accept and trust this new technology [6, 7] and contributes to enroll candidates for AIDs who are motivated and with proper expectations [6, 7].

According to the Technology Acceptance Model (TAM), analysis of youths and parents’ attitudes towards a technology device, including AIDs, should evaluate the following aspects: intention to use (as a measure of future acceptance), perceived usefulness, ease of use, and trust. Semi-structured interviews based on the TAM and different questionnaires to assess expected AIDs acceptance have been developed [8–13] and have allowed diabetes care providers to tailor their education approach to the factors that concern the patient at that time, and to implement behavioral strategies supporting sustained use of the AID system [14].

However, these questionnaires have only been validated in English, and when used in the Italian population, the questions were translated with the assistance of native English speakers but not validated [10]. Therefore, this study aimed to create a questionnaire in Italian for expected AIDs acceptance based on the TAM model, starting from the questionnaire items available in the literature.

## Subjects and methods

### Development of the AP acceptance questionnaire

#### Step 1: literature review

A questionnaire review on the topic was performed in the literature, and the results were reported in a recently published paper [14]. We analyzed questionnaire items reported in previously published studies. We evaluated their recurrence as reported in Supplementary material S1 [9–13, 15], and we classified open and closed questions according to the TAM: (1) Intention to use; (2) Perceived usefulness and its determinants; (3) Perceived ease of use and its determinants; (4) Trust in the AP (Supplementary material S1).

#### Step 2: interview of patients and their caregivers

The pediatric diabetologists prepared a written introduction to the AID technology (RF, MM, EM), explaining CSII and CGM characteristics and the algorithm integrated into the pump, alarms, and safety issues (Supplementary material S2). The name “artificial pancreas” was preferred in the introduction and the questionnaire, as it is the most used term among our patients.

The open and closed questions collected during the analysis of measures available in the literature were used by our psychologists (SZ, DC, AA) as a topic guide to perform interviews addressed to 15 subjects with T1D followed up at three different centers (Trento, Verona and Chieti), aged 8–18 years, with diabetes duration > 1 year, on multiple daily injections (MDI) or a sensor-augmented insulin pump (SAP) from at least six months, and to their parents.

The psychologists administered the interview after the written introduction to AIDs was presented (Supplementary material S2), and a prototype was visualized.

#### Step 3: writing of the closed questions

The open questions used during the interview were converted to closed questions and used with those derived from the interview. We divided these questions into five domains, adding a new one called “judgment by others” (or subjective norms) to the four ones proposed by the TAM model: (1) Intention to use; (2) Perceived usefulness and its determinants; (3) Judgement by others; (4) Perceived ease of use and its determinants; (5) Trust in the AP.

A total of 43 questions for children and 46 for parents were included, and a 5-point Likert scale was used. 1 = strongly disagree, 2 = disagree, 3 = neutral, 4 = agree, 5 = strongly agree. The parents’ measure included three more items compared to the patients’ one: an item on the expectation to reduce severe hypoglycemia (item #8), another to reduce nocturnal hypoglycemia (item #9), and one about the increased risk of ketoacidosis (#46). The first version, 1.0, of the questionnaire was developed.

### Measure testing on a pilot sample (cognitive debriefing)

To evaluate if the questions were formulated, version 1.0 of the questionnaire was tested on a small sample of the target population: 5 patients and their parents. The psychologists (SZ, CD, AA) asked the patients whether there were clarity issues, culturally inappropriate expressions, or difficulties in understanding the questions. The debriefing interviews involved paraphrasing each questionnaire question and indicating whether the participants needed help understanding the question or if any terms required clarification. Subsequently, the scientific panel discussed the feedback from the five patients. The panel accepted some proposals, and two sentences were rephrased; a new version based on the issues raised was developed (version 1.1, Supplementary materials 3 and 4).

### Validation of the questionnaire

The Clinical Research Ethics Committee of the coordinating center of Trento approved the study (A930, AP-acceptance), which followed the Declaration of Helsinki. Written informed assents and consents were obtained by minors aged ≥ 12 and all parents before study entry. The pediatric diabetes centers participating in the study belonged to the Italian Society for Pediatric Endocrinology and Diabetes [16]. The involved centers were Trento, Verona, Napoli Federico II, Bologna, Novara, Genova, Messina, Ancona, and Chieti. The questionnaire was administered by the pediatric diabetologist and/or psychologist to all the patients who attended the centers in the period from September to December 2023 and who met the following inclusion criteria:

- T1D, aged 8 to 18 years: we considered the lower limit of 8 years for understanding the questions, as previously reported in questionnaires like INSPIRE [13];
- diabetes duration ≥ 12 months;
- on multiple daily injections (MDI) or SAP (CSII and real-time or instant scanning CGM) for at least six months;
- HbA1c < 10%.
- fluent in Italian, evaluated as being able to express and read easily.

**The exclusion criteria** were:

- subjects on CSII with predictive low glucose monitoring (PLGM), hybrid closed loop (HCL), or advanced hybrid closed loop (AHCL);
- complications related to T1D or other significant diseases or comorbidities.

### Statistical analysis

Analyses were conducted using SAS v9.1.4. (SAS Institute Inc., Cary, NC). Items were measured using five-category scales. Considering the single items as quantitative variables, means, standard deviations (SD), and medians were calculated separately for young people and parents. Factor analysis used minimum residuals on the correlation matrix approach to determine the model best describing the data, always separately for young people and parents. For choosing the number of factors, eigenvalues ≥ 1 were the criterion. A sample size of at least 220 participants was sufficient to perform factor analysis, including at least five cases per item. Cronbach’s α coefficient (α = k x r /[1 + (k − 1) x r]; with k = number of items and r = mean correlation) was calculated for each item and, for the total of the items, keeping only the records for which all answers relating to each section were present. The aggregating dimensions of the AP future acceptance Italian version were evaluated by factor analysis (principal components). Furthermore, Spearman correlations between each item and all other items were calculated to identify any significant correlations between pairs of variables, with a correlation coefficient > 0.70 considered vital [17]. Finally, agreement between young people and parent scores was calculated using Gwet’s agreement coefficient (AC1 with 95% confidence intervals (CI)), considered more stable than Cohen’s kappa.

All variables are presented as frequencies, percentages, mean ± SD, and medians. The Kolmogorov-Smirnov test was used to verify the normality of distributions. The mean and SD of the item scores were calculated. The significance level was set to a *p*-value ≤ 0.05.

The overall score on the questionnaire for patients and parents will be calculated by obtaining a mean score across items for each subject after the reverse score calculation of items classified as hassles. Higher scores will indicate greater positive expectations for AIDs. The overall score on benefit and hassles items will be calculated by obtaining a mean score across items classified in each of the two categories.

## Results

In total, 254 parents (182 mothers, 71.6%) and 254 children-adolescents (113 female, 55.5%) were recruited for the study (*p* < 0.00001). Fifteen couples of questionnaires were excluded because the anonymized code was misreported, and we could not match the pairs. Therefore, 239 questionnaire couples were analyzed for this study, and the subjects’ characteristics are shown in Table 1.

**Table 1:** Characteristics of subjects with T1D enrolled in the study. Data are reported as [mean ± SD (median)]. CGM: continuous glucose monitoring, MDI: multiple daily injections, SAP: sensor-augmented pump, BMI: body mass index. HbA1c: glycosylated hemoglobin. TIR: time in range

*N* = 239	
**Female n (%)**	113 (47.3)
**Age at study enrollment (years)** [mean ± SD (Median)]	14.21 ± 2.59 (14.56)
**Age at diabetes onset (years)** [mean ± SD (Median)]	6.91 ± 3.82 (7.07)
**Age at CGM start** [mean ± SD (Median)]	9.59 ± 3.29 (9.72)
**CGM experience (years)** [mean ± SD (Median)]	4.61 ± 2.13 (4.44)
**CGM type n (%)**	
Dexcom G6 Free Style Libre 2 Free Style Libre 3	155 (64.8) 79 (33.0) 5 (2.1)
**Insulin treatment n (%)**	
MDI SAP	72 (30.1) 167 (69.9)
**Weight (Kg)** [mean ± SD (Median)]	54.2 ± 14.5 (55.9)
**Height (m)** [mean ± SD (Median)]	159.9 ± 13.9 (161.4)
**BMI z-score**	0.34 ± 0.94 (0.30)
**Stage of Puberty n (%)**	
Prepubertal Pubertal Postpubertal	37 (15.5) 60 (25.1) 142 (59.4)
**% HbA1c annual** [mean ± SD (Median)] **HbA1c annual** [mean ± SD (Median)] in mmol/mol	7.1 ± 0.8 (7.0) 54 ± 15 (53)
**% HbA1c last value** [mean ± SD (Median)] **HbA1c annual** [mean ± SD (Median)] in mmol/mol	7.2 ± 0.9 (7.2) 55 ± 14 (55)
**Total daily insulin dose (U/Kg** [mean ± SD (Median)]	0.77 ± 0.28 (0.78)
**% of time with active sensor** [mean ± SD (median)]	89.3 ± 14.6 (95.0)
**% of time in range (70–180 mg/dL)** [mean ± SD (median)]	59.4 ± 15.6 (60.0)
**% of time below range < 70 mg/dL** [mean ± SD (median)]	2.9 ± 2.8 (2.0)
**% of time below range < 54 mg/dL** [mean ± SD (median)]	0.8 ± 1.3 (0.0)
**% of time above range > 180 mg/dL** [mean ± SD (median)]	26.1 ± 12.4 (24.0)
**% of time above range > 250 mg/dL** [mean ± SD (median)]	12.8 ± 11.6 (9.0)
**Mean glucose (mg/dL)** [mean ± SD (median)]	166.6 ± 29.3 (160.5)
**% Coefficient of variation (CV)** [mean ± SD (median)]	37.6 ± 7.0 (37.0)
**% Glucose management indicator (GMI)** [mean ± SD (median)]	7.32 ± 0.74 (7.3)

### Reliability and factor analysis

#### Evaluation of completeness

Completeness was optimal for each item for subjects with T1D and parents (i.e., the maximum percentage of missing values was 0.4 in the group of subjects with T1D (only for item n. 42); the parents’ group reached 100% of items answered).

#### Internal reliability

Cronbach’s coefficients > 90% were recorded for all 43 items in the children-adolescents group and all 46 items in the parents’ group. The overall Cronbach’s coefficients were 0.92 and 0.93, indicating excellent internal consistency in both groups.

#### Factor analysis

For the AP measure, two factors emerged, both in the group of children-adolescents and in that of the parents: Benefits of AIDs (30 items for children-adolescents and 32 for parents) and hassles of AIDs (10 items for children-adolescents and 11 for parents). In both groups, the same remaining three items did not load on any factor (“*I don’t think AIDs will be useful if with tube*”, *“I think it will not be simple to learn, but time by time it could be easier”*, *“I think I will need a training course before using AIDs”* Table 2A). After the three items that did not load on a factor were deleted, the internal consistency of the total scale was alpha = 0.94 for subjects with T1D and 0.95 for parents.

**Table 2A:** Results of the factor analysis for the Artificial pancreas. Y: children-adolescents, p: parents

Measurement factor	Items loading	Eigenvalue	% variance	Alpha coefficient
Benefits of Artificial Pancreas	Item numbers: 1, 2, 4, 5, 6, 7, 8, 9, 10, 11, 13, 14, 19, 20, 22, 25, 26, 28, 29, 30, 31, 32, 33, 34, 35, 36, 37, 39, 40, 41 (y) Item numbers: 1, 2, 4, 5, 6, 7, 8, 9, 10, 11, 12, 13, 15, 16, 21, 22, 24, 27, 28, 30, 31, 32, 33, 34, 35, 36, 37, 38, 39, 41, 42, 43 (p)	13.37 (y) 14.82 (p)	46.2% (y) 45.5% (p)	0.95 (y) 0.96 (p)
Hassles of Artificial Pancreas	Item numbers: 3, 12, 16, 17, 18, 21, 23, 24, 42, 43 (y) Item numbers: 3, 14, 18, 19, 20, 23, 25, 26, 44, 45, 46 (p)	3.92 (y) 4.55 (p)	13.5% (y) 140% (p)	0.84 (y) 0.85 (p)
No factor	Item numbers: 15, 27 and 38 (y) Item numbers: 17, 29 and 40 (p)			

#### Correlation analysis

The correlation analysis showed positive correlations between couples of items greater than 0.30 in many cases but never greater than 0.80. Even if some items are correlated to each other, they do not necessarily need to be eliminated because they have a specific interest. The three items that did not load on any factor are those that show the lowest correlation coefficients.

#### Agreement analysis

The Gwet’s agreement coefficient (AC1) was 0.699 (95% C.I. 0.689– 0.709), indicating a good agreement between the two evaluations (children-adolescents and parents). The descriptive statistics and reliability indices for the Artificial Pancreas for children-adolescents and parents are summarized in Table 2B.

**Table 2B:** Descriptive statistics and reliability indices for the artificial pancreas for children and parents

	Children	Parents
*N*	239	239
Mean ± SD item score	3.9 ± 1.0	3.9 ± 1.0
Alpha coefficient	0.92	0.93
Parent–children agreement AC1 (95% C.I.)	0.699 (0.689– 0.709)	

Before the overall score calculation, the reverse score was calculated using the measures of patients and parents for items classified as hassles. The level of AP acceptance was more than neutral, as indicated by the mean overall score of 3.91 ± 0.47 and 3.99 ± 0.43 (*p* = 0.07) for youths and parents, respectively (possible score range 1 to 5, with a neutral score of 3.0). Parents reported higher scores in the benefit items than children-adolescents (*p* = 0.04).

## Discussion

Through this study, we developed a new questionnaire based on the items available in the literature [9–13] and used in studies on AID acceptance [18–24]. We demonstrated that the AP-acceptance questionnaire is a reliable and valid measure of expectancies regarding AIDs by subjects with T1D and their parents. It reveals a meaningful factor structure, good internal reliability, and agreement between parents and young people in evaluations.

It consists of 43 items in the children-adolescents group and 46 in the parents’ group. In line with previous validated AP acceptance questionnaires [9, 11], two primary measurement factors were isolated: benefits of AP (30 (y), 32 (p) items) and hassles of AP (10 (y), 11 (p) items), while three items (15, 27 and 38 in youths; 17, 29 and 40 in parents) did not load on any measurement factor and showed the lowest correlation coefficients. However, a 40 and 43-item questionnaire without the three items would not change the internal consistency of the questionnaire.

This questionnaire measured positive expectancies of what an AID can do, and above all, expected improvements in glycemic control and diabetes-specific well-being emerged from youths and their parents. As previously reported, intention to use AIDs could be considered a measure of future acceptance, and the level of AP acceptance was more than neutral [9]. Mean scores on the “benefits” were higher for patients/parents than “hassles”, indicating they had a positive attitude toward perceived usefulness, ease of use, and trust.

This study presents the following strengths:

1) All the patients enrolled were familiar with CGM, as in Italy, subjects with T1D receive reimbursement for this device, differently from studies set in other countries [9]; therefore, conclusions of this study are generalizable to all the population we face in our offices; subjects enrolled in our study acknowledged the performance of this component of AIDs, and this was reflected in a more critical approach to the AIDs;

2) Even if individuals with SAP could be the most likely first candidates for AP systems, and they better know the limits of CSII and its influence on body perception, we decided to include not only subjects with T1D who were treated with CSII but also children and young people on MDI therapy, to avoid population bias selection, unlike previous reports [9].

The main limitation considered in interpreting the results of our study is the cross-sectional design, and future studies should evaluate correlations between AP scores and patients’ and/or technology characteristics.

In conclusion, our study provided the first validated questionnaire in Italian for AP acceptance. It could be a valuable resource for clinicians and researchers to assess AP acceptance in pediatric patients with T1D and their parents. This patient profiling approach could help to enroll candidates for AIDs with proper expectations and who most likely will benefit from the system.

## Electronic supplementary material

Below is the link to the electronic supplementary material.

Supplementary Material 1

Supplementary Material 2

Supplementary Material 3

Supplementary Material 4

## Data Availability

All databases generated for this study are included in the article.

## Abbreviations

AHCL: Advanced hybrid closed loop
AID: Automated insulin delivery
AP: Artificial pancreas
CGM: Continuous glucose monitoring
CSII: Continuous subcutaneous insulin infusion
HCL: Hybrid closed loop
MDI: Multiple daily injections
PLGM: Predictive low glucose monitoring
SAP: Sensor-augmented pump
SD: Standard deviation
T1D: Type 1 diabetes
TAM: Technology Acceptance Model
