# Supplementary material for: Future acceptance of automated insulin delivery systems in youths with type 1 diabetes: validation of the Italian artificial pancreas-acceptance measure
Source: Acta Diabetol. 2024 Aug 10;62(2):177–83. doi: 10.1007/s00592-024-02327-9 (PMC11861114; doi:10.1007/s00592-024-02327-9)
Supplement: Supplementary file 1 — Supplementary Material 1 [file 592_2024_2327_MOESM1_ESM.docx]

**Table S1:** Questionnaire items and their recurrence in previously published measures on AID future acceptance, in youths (1A) and their parents (1B).

**1A**

| **INTERVISTA SEMISTRUTTURATA** dopo introduzione  su “Che cos’è il pancreas artificiale” | **QUESTIONARIO SULLE ASPETTATIVE PER PAZIENTI**  (8-18 anni)   (0=strongly disagree, 1=moderately disagree, 2=somewhat disagree, 3=neutral, 4=somewhat agree, 5=moderately agree, and 6=strongly agree) | Van Bon AC et al.  2011  (Adult) | Troncone A et al. 2016 | Bevier WC et al. 2014 | Oukes T et al. 2019 | Naranjo J et al. 2016 (DSAT) | Weissberg-Benchell J et al. 2019 (INSPIRE) |
| --- | --- | --- | --- | --- | --- | --- | --- |
| In generale ti piacerebbe il pancreas artificiale? Se si, per che cosa? | **1-Intenzione all’utilizzo di AP** |  | | | | | |
| Pensi che l’utilizzo del pancreas artificiale sia utile? Se si, perché? | C- Mi piacerebbe provare la terapia con pancreas artificiale | X | X | X |  |  | X |
| Pensi che l’utilizzo del pancreas artificiale porti dei problemi? Se si, quali? | C- Mi piacerebbe utilizzare il pancreas artificiale per un lungo periodo | X | X |  |  |  |  |
| Pensi che l’utilizzo del pancreas artificiale renda più semplice controllare il diabete? | **2-Percezione dell’utilità** in relazione al controllo glicemico |  | | | | | |
|  | C-il pancreas artificiale migliorerà il mio controllo del glucosio e l’emoglobina glicata | X | X |  |  |  | X |
|  | C-il pancreas artificiale ridurrà il numero di ipoglicemie | X | X | X |  |  | X |
|  | C-il pancreas artificiale ridurrà il numero di iperglicemie | X | X | X |  |  | X |
|  | C-il pancreas artificiale ridurrà le mie preoccupazioni per il diabete | X | X |  |  |  | X |
|  | C-il pancreas artificiale ridurrà le preoccupazioni della mia famiglia per il diabete |  |  |  |  |  | X |
|  | C-il pancreas artificiale renderà la mia vita più semplice | X | X |  | X | X | X |
|  | **Determinanti** dell’utilità percepita |  | | | | | |
|  | C- il pancreas artificiale mi farà dedicare meno tempo della mia giornata al diabete | X | X |  |  | X | X |
|  | C- il pancreas artificiale ridurrà la frequenza delle visite con il medico | X |  | X |  |  |  |
|  | C- il pancreas artificiale ridurrà la frequenza delle visite con l’infermiere | X |  |  |  |  |  |
| Secondo te, cosa diranno i tuoi insegnanti se tu utilizzerai il pancreas artificiale? | C- le persone che sono per me importanti (famiglia e amici), saranno d’accordo che mi serva utilizzare il pancreas artificiale | X | X |  | X |  |  |
| Secondo te, cosa diranno i tuoi compagni di classe e amici se tu utilizzerai il pancreas artificiale? | C- indossare il pancreas artificiale mi renderà un esempio verso le altre persone con diabete | X | X |  |  |  |  |
|  | C- Indossare il pancreas artificiale in pubblico mi farà sentire diverso | X |  |  |  |  |  |
|  | **3-Percezione della semplicità di utilizzo** |  | | | | | |
| Pensi che il pancreas artificiale sia semplice da utilizzare? | C- il pancreas artificiale mi sembra semplice da utilizzare | X | X | X |  |  |  |
| Pensi che altri pazienti con diabete potrebbero essere in grado di utilizzare il pancreas artificiale? | C-il pancreas artificiale migliorerà la mia qualità di vita |  |  | X | X | X |  |
|  | C-il pancreas artificiale migliorerà la qualità di vita della mia famiglia |  |  |  |  |  | X |
|  | C- il pancreas artificiale renderà più facile l’alimentazione |  |  |  | X |  | X |
|  | C- il pancreas artificiale renderà più facile l’esercizio fisico |  |  |  | X |  | X |
|  | C- il pancreas artificiale renderà più facile la gestione del diabete a scuola o al lavoro |  |  |  | X |  | X |
|  | C- il pancreas artificiale renderà più facile la gestione del diabete durante i giorni di malattia |  |  |  |  |  | X |
|  | C- il pancreas artificiale mi farà dormire meglio |  |  |  |  |  | X |
|  | **Determinanti della semplicità all’utilizzo** |  | | | | | |
| Sarai in grado di utilizzare da solo il pancreas artificiale o ti servirà l’aiuto dei genitori? | C- penso che sarò in grado di utilizzare bene il pancreas artificiale | X | X |  |  |  |  |
|  | C- mi servirà un corso prima di utilizzare il pancreas artificiale | X |  |  |  |  |  |
|  | **4-Fiducia** nell’AP |  | | | | | |
| Sei fiducioso che il pancreas artificiale può controllare il diabete? | C-ho fiducia che il pancreas artificiale somministrerà la quantità corretta di insulina | X | X |  |  |  |  |
|  | C-ho fiducia delle misurazioni del glucosio che effettua il pancreas artificiale | X | X |  |  |  |  |
|  | C-gli allarmi aiuteranno la mia fiducia nel pancreas artificiale | X |  |  |  |  |  |

**1B**

| **INTERVISTA SEMISTRUTTURATA** dopo introduzione su che “Cos’è il pancreas artificiale” | **QUESTIONARIO SULLE ASPETTATIVE PER GENITORI** | Van Bon AC et al.  2011 | Troncone A et al. 2016 | Bevier WC et al. 2014 | Oukes T et al. 2019 | Naranjo J et al. 2016 (DSAT) | Weissberg-Benchell J et al. 2019 (INSPIRE) |
| --- | --- | --- | --- | --- | --- | --- | --- |
| In generale ti piace il pancreas artificiale? Se si, che cosa? | **1-Intenzione all’utilizzo** | | | | | | |
| Quali possibili vantaggi prevedi? | P- Mi piacerebbe che mio figlio/a provasse la terapia con pancreas artificiale |  | X |  |  |  | X |
| Quali svantaggi prevedi? | P- Mi piacerebbe che mio figlio/a utilizzasse il pancreas artificiale per un lungo periodo |  | X |  |  |  |  |
| Pensi che l’utilizzo del pancreas artificiale migliori il controllo del glucosio? | **2-Percezione dell’utilità** in relazione al controllo glicemico |  |  |  |  |  |  |
|  | P-il pancreas artificiale migliorerà il controllo del glucosio e l’emoglobina glicata di mio figlio/a |  | X |  |  |  | X |
|  | P-il pancreas artificiale ridurrà il numero di ipoglicemie di mio figlio/a |  | X |  |  |  | X |
|  | P-il pancreas artificiale ridurrà il numero di iperglicemie di mio figlio/a |  | X |  |  |  | X |
|  | P-il pancreas artificiale ridurrà le preoccupazioni della nostra famiglia riguardo al diabete |  | X |  |  |  | X |
|  | P-il pancreas artificiale renderà la vita di mio figlio/a più semplice |  | X |  |  |  | X |
|  | **Determinanti dell’utilità del pancreas artificiale** | | | | | | |
| Secondo te, cosa diranno gli insegnanti di tuo figlio/a se utilizzerà il pancreas artificiale? | P- il pancreas artificiale mi farà dedicare meno tempo della mia giornata al diabete di mio figlio/a |  | X |  |  |  | X |
|  | P- il pancreas artificiale ridurrà la frequenza delle visite con il medico |  |  |  |  |  |  |
|  | P- il pancreas artificiale ridurrà la frequenza delle visite con l’infermiere |  |  |  |  |  |  |
| Secondo te, cosa diranno i compagni di classe e amici di tuo figlio/a se utilizzerà il pancreas artificiale? | P- le persone che sono per me importanti (famiglia e amici), saranno d’accordo che serva utilizzare il pancreas artificiale per mio figlio/a |  | X |  |  |  |  |
|  | P- indossare il pancreas artificiale renderà mio figlio/a un esempio verso le altre persone con diabete |  | X |  |  |  |  |
|  | P- Indossare il pancreas artificiale in pubblico renderà mio figlio/a più insicuro/a |  |  |  |  |  |  |
|  | **3-Percepito come semplice da utilizzare** | | | | | | |
| Pensi che il pancreas artificiale sia semplice da utilizzare? | P- il pancreas artificiale sembra semplice da utilizzare |  | X |  |  |  |  |
| Pensi che altri pazienti con diabete potrebbero essere in grado di utilizzare il pancreas artificiale? | P-il pancreas artificiale migliorerà la mia qualità di vita |  |  |  |  |  | X |
|  | P-il pancreas artificiale migliorerà la qualità di vita della mia famiglia |  |  |  |  |  | X |
|  | P- il pancreas artificiale renderà più facile l’esercizio fisico |  |  |  |  |  | X |
|  | P- il pancreas artificiale renderà più facile la gestione del diabete a scuola o al lavoro |  |  |  |  |  | X |
|  | P- il pancreas artificiale renderà più facile la gestione del diabete durante i giorni di malattia |  |  |  |  |  | X |
|  | P- il pancreas artificiale mi farà dormire meglio |  |  |  |  |  | X |
|  | **Determinanti della semplicità all’utilizzo** | | | | | | |
| Pensi che tuo figlio/a sarà in grado di utilizzare da solo il pancreas artificiale o gli/le servirà l’aiuto dei genitori? | P- penso che mio figlio sarà in grado di utilizzare bene il pancreas artificiale |  | X |  |  |  |  |
|  | P-mi servirà un corso prima di utilizzare il pancreas artificiale |  |  |  |  |  |  |
|  | **4-Fiducia** nell’AP | | | | | | |
| Sei fiducioso che il pancreas artificiale può controllare il diabete? | P-ho fiducia che il pancreas artificiale somministrerà la quantità corretta di insulina |  | X |  |  |  |  |
|  | P-ho fiducia delle misurazioni del glucosio del pancreas artificiale |  | X |  |  |  |  |
|  | P-gli allarmi aiuteranno la mia fiducia nel pancreas artificiale |  |  |  |  |  |  |
