# Supplementary material for: Future acceptance of automated insulin delivery systems in youths with type 1 diabetes: validation of the Italian artificial pancreas-acceptance measure
Source: Acta Diabetol. 2024 Aug 10;62(2):177–83. doi: 10.1007/s00592-024-02327-9 (PMC11861114; doi:10.1007/s00592-024-02327-9)
Supplement: Supplementary file 2 — Supplementary Material 2 [file 592_2024_2327_MOESM2_ESM.docx]

**Supplementary material S2: introduction to AID systems**

Il pancreas artificiale è l’insieme di
- un sensore che misura in continuo il valore di glucosio nel liquido tra le cellule;
- un microinfusore che infonde insulina nel sottocute;

- un algoritmo, cioè delle informazioni contenute nel microinfusore o in un telefono, che decide la quantità di insulina da infondere nel sottocute in base al valore di glucosio registrato dal sensore in quel momento e alla previsione di quanto accadrà nelle prossime ore.

Questo sistema non è ancora come il vero pancreas, perché l’insulina è rilasciata in ritardo rispetto a quello che farebbe il pancreas e non la infonde in una vena ma nel sottocute. Inoltre il sensore misura il valore del glucosio nel liquido tra le cellule, e non nei vasi sanguigni, e quindi anche questo è in ritardo.

Il pancreas artificiale evita di iniettarsi l’insulina più volte al giorno e di doversi controllare la glicemia più volte al giorno. Evita inoltre che ci siano picchi di glicemia o che la glicemia si abbassi troppo, regolando da solo l’insulina necessaria. Il pancreas artificiale presenta degli allarmi di sicurezza che suonano nel caso il sistema non riesca a gestire valori troppo alti o troppo bassi di glucosio.

Ad oggi tutti i sistemi richiedono ancora che al pasto venga inserito quanti carboidrati si stanno per mangiare e poi si devono confermare le quantità di insulina calcolate dal pancreas artificiale. Inoltre i tempi di attesa vanno rispettati anche con questo sistema.

È richiesto che vengano indossati due dispositivi (microinfusore e sensore) e che vengano sostituiti con regolarità, prima in caso di malfunzionamento.
