# Supplementary material for: Future acceptance of automated insulin delivery systems in youths with type 1 diabetes: validation of the Italian artificial pancreas-acceptance measure
Source: Acta Diabetol. 2024 Aug 10;62(2):177–83. doi: 10.1007/s00592-024-02327-9 (PMC11861114; doi:10.1007/s00592-024-02327-9)
Supplement: Supplementary file 3 — Supplementary Material 3 [file 592_2024_2327_MOESM3_ESM.docx]

**Allegato 1: AP-Acceptance questionario 1.1. Genitori**

Cerchia il numero accanto ad ogni affermazione che più si avvicina alla tua opinione.

Cerchia solo un numero e non lasciare niente in bianco

|  | In completo disaccordo | In disaccordo | Neutrale | | D’accordo | | Completamente d’accordo |
| --- | --- | --- | --- | --- | --- | --- | --- |
| **Cosa ne pensi di un futuro utilizzo del pancreas artificiale per tuo figlio/a…** | | | | | | | |
| 1) Mi piacerebbe che mio figlio/a lo provasse | 1 | 2 | 3 | 4 | | 5 | |
| 2) Mi piacerebbe che mio figlio/a lo utilizzasse per un lungo periodo | 1 | 2 | 3 | 4 | | 5 | |
| 3) Lo/la renderà ancora più diverso | 1 | 2 | 3 | 4 | | 5 | |
| **Pensi sia utile per il controllo della glicemia?** | | | | | | | |
| 4) Migliorerà il controllo del glucosio, il time in range e l’emoglobina glicata di mio figlio/a | 1 | 2 | 3 | 4 | | 5 | |
| 5) Ridurrà il numero di ipoglicemie | 1 | 2 | 3 | 4 | | 5 | |
| 6) Ridurrà il numero di iperglicemie | 1 | 2 | 3 | 4 | | 5 | |
| 7) Ridurrà il rischio di complicanze | 1 | 2 | 3 | 4 | | 5 | |
| 8) Ridurrà il rischio di ipoglicemia grave | 1 | 2 | 3 | 4 | | 5 | |
| 9) Ridurrà il rischio di ipoglicemie notturne | 1 | 2 | 3 | 4 | | 5 | |
| 10) Ridurrà le preoccupazioni di mia figlia/o per il diabete | 1 | 2 | 3 | 4 | | 5 | |
| 11) Ridurrà le preoccupazioni della mia famiglia per il diabete | 1 | 2 | 3 | 4 | | 5 | |
| **Come il pancreas artificiale può essere utile** | | | | | | | |
| 12) Mi farà dedicare meno tempo della giornata al diabete di mio figlio/a | 1 | 2 | 3 | 4 | | 5 | |
| 13) Non dovrà più usare le penne, se non raramente | 1 | 2 | 3 | 4 | | 5 | |
| 14) Temo perda la costanza dei controlli | 1 | 2 | 3 | 4 | | 5 | |
| 15) Le/gli permetterà un’alimentazione con meno rinunce | 1 | 2 | 3 | 4 | | 5 | |
| 16) Avrà meno preoccupazioni durante il gioco o lo sport | 1 | 2 | 3 | 4 | | 5 | |
| 17) Non credo sia utile se ha il tubicino | 1 | 2 | 3 | 4 | | 5 | |
| 18) Potrebbe modificare l’immagine che ha del suo corpo | 1 | 2 | 3 | 4 | | 5 | |
| 19) Avere due dispositivi attaccati al suo corpo potrebbe darle/gli fastidio | 1 | 2 | 3 | 4 | | 5 | |
| 20) Avere due dispositivi attaccati al corpo la/lo potrebbe limitare in alcune attività quotidiane | 1 | 2 | 3 | 4 | | 5 | |
| **Secondo te cosa diranno gli altri se utilizzerà il pancreas artificiale?** | | | | | | | |
|  | In completo disaccordo | In disaccordo | Neutrale | D’accordo | | Completamente d’accordo | |
| 21) Le persone che sono per me importanti (famiglia e amici), saranno d’accordo che serva utilizzare il pancreas artificiale per mio figlio/a | 1 | 2 | 3 | 4 | | 5 | |
| 22) Gli insegnanti la/lo coinvolgeranno nella condivisione della novità con la classe | 1 | 2 | 3 | 4 | | 5 | |
| 23) In classe sarà sicuramente un problema perché i compagni non amano le novità | 1 | 2 | 3 | 4 | | 5 | |
| 24) I compagni non diranno nulla, saranno solamente interessati in un primo momento | 1 | 2 | 3 | 4 | | 5 | |
| 25) I compagni potrebbero porre domande con insistenza | 1 | 2 | 3 | 4 | | 5 | |
| 26) Altre persone potrebbero guardarla/lo con insistenza | 1 | 2 | 3 | 4 | | 5 | |
| 27) Renderà mio figlio/a un esempio verso le altre persone con diabete | 1 | 2 | 3 | 4 | | 5 | |
| **Pensi che il pancreas artificiale sia semplice da utilizzare?** | | | | | | | |
| 28) Penso sia semplice da utilizzare | 1 | 2 | 3 | 4 | | 5 | |
| 29) Penso non sarà facile imparare, ma con il tempo potrebbe essere più semplice | 1 | 2 | 3 | 4 | | 5 | |
| 30) Potrebbe migliorare la qualità di vita di mia figlia/o | 1 | 2 | 3 | 4 | | 5 | |
| 31) Potrebbe migliorare la qualità di vita della mia famiglia | 1 | 2 | 3 | 4 | | 5 | |
| 32) Potrebbe rendere più facile l’alimentazione | 1 | 2 | 3 | 4 | | 5 | |
| 33) Potrebbe rendere più facile l’esercizio fisico e lo sport | 1 | 2 | 3 | 4 | | 5 | |
| 34) Permetterà a mia figlia/o di essere più indipendente | 1 | 2 | 3 | 4 | | 5 | |
| 35) Renderà più facile la gestione del diabete a scuola o al lavoro | 1 | 2 | 3 | 4 | | 5 | |
| 36) Renderà più facile la gestione del diabete durante i giorni di malattia | 1 | 2 | 3 | 4 | | 5 | |
| 37) La/lo farà dormire meglio | 1 | 2 | 3 | 4 | | 5 | |
| 38) Permetterà a noi genitori di dormire di più | 1 | 2 | 3 | 4 | | 5 | |
| 39) Penso che mia figlia/o sarà in grado di utilizzare bene il pancreas artificiale | 1 | 2 | 3 | 4 | | 5 | |
| 40) Penso mi servirà un corso prima di utilizzare il pancreas artificiale | 1 | 2 | 3 | 4 | | 5 | |
| **Sei fiducioso sul funzionamento del pancreas artificiale?** | | | | | | | |
|  | In completo disaccordo | In disaccordo | Neutrale | D’accordo | | Completamente d’accordo | |
| 41) Ho fiducia delle misurazioni del glucosio che effettua il pancreas artificiale | 1 | 2 | 3 | 4 | | 5 | |
| 42) Ho fiducia che il pancreas artificiale somministrerà la quantità corretta di insulina | 1 | 2 | 3 | 4 | | 5 | |
| 43) Gli allarmi aiuteranno la mia fiducia nel pancreas artificiale | 1 | 2 | 3 | 4 | | 5 | |
| 44) Gli allarmi mi potrebbero disturbare nell’attività quotidiana | 1 | 2 | 3 | 4 | | 5 | |
| 45) Gli allarmi mi potrebbero disturbare soprattutto la notte | 1 | 2 | 3 | 4 | | 5 | |
| 46) Il pancreas artificiale aumenta il rischio di chetoacidosi | 1 | 2 | 3 | 4 | | 5 | |
