# Supplementary material for: Future acceptance of automated insulin delivery systems in youths with type 1 diabetes: validation of the Italian artificial pancreas-acceptance measure
Source: Acta Diabetol. 2024 Aug 10;62(2):177–83. doi: 10.1007/s00592-024-02327-9 (PMC11861114; doi:10.1007/s00592-024-02327-9)
Supplement: Supplementary file 4 — Supplementary Material 4 [file 592_2024_2327_MOESM4_ESM.docx]

**Allegato 1: AP-Acceptance questionario 1.1. Bambini > 8 anni e adolescenti**

Cerchia il numero accanto ad ogni affermazione che più si avvicina alla tua opinione.

Cerchia solo un numero e non lasciare niente in bianco

|  | In completo disaccordo | In disaccordo | Neutrale | D’accordo | Completamente d’accordo |
| --- | --- | --- | --- | --- | --- |
| **Cosa ne pensi di un tuo futuro utilizzo del pancreas artificiale?** | | | | | |
| 1) Mi piacerebbe provarlo | 1 | 2 | 3 | 4 | 5 |
| 2) Mi piacerebbe utilizzarlo per un lungo periodo | 1 | 2 | 3 | 4 | 5 |
| 3) Mi renderà ancora più diverso | 1 | 2 | 3 | 4 | 5 |
| **Pensi sia utile per il controllo della glicemia?** | | | | | |
| 4) Migliorerà il mio controllo del glucosio, il time in range, e l’emoglobina glicata | 1 | 2 | 3 | 4 | 5 |
| 5) Ridurrà il numero di ipoglicemie | 1 | 2 | 3 | 4 | 5 |
| 6) Ridurrà il numero di iperglicemie | 1 | 2 | 3 | 4 | 5 |
| 7) Ridurrà il rischio di complicanze | 1 | 2 | 3 | 4 | 5 |
| 8) Ridurrà le mie preoccupazioni per il diabete | 1 | 2 | 3 | 4 | 5 |
| 9) Ridurrà le preoccupazioni della mia famiglia per il diabete | 1 | 2 | 3 | 4 | 5 |
| **Come il pancreas artificiale può esserti utile** | | | | | |
| 10) Mi farà dedicare meno tempo della giornata al diabete | 1 | 2 | 3 | 4 | 5 |
| 11) Non dovrò più usare le penne, se non raramente | 1 | 2 | 3 | 4 | 5 |
| 12) Temo di perdere la costanza dei controlli | 1 | 2 | 3 | 4 | 5 |
| 13) Mi permetterà un’alimentazione con meno rinunce | 1 | 2 | 3 | 4 | 5 |
| 14) Avrò meno preoccupazioni durante il gioco o lo sport | 1 | 2 | 3 | 4 | 5 |
| 15) Non credo sia utile se ha il tubicino | 1 | 2 | 3 | 4 | 5 |
| 16) Potrebbe modificare l’immagine che ho del mio corpo | 1 | 2 | 3 | 4 | 5 |
| 17) Avere due dispositivi attaccati al mio corpo potrebbe darmi fastidio | 1 | 2 | 3 | 4 | 5 |
| 18) Avere due dispositivi attaccati al corpo mi potrebbe limitare in alcune attività quotidiane | 1 | 2 | 3 | 4 | 5 |
|  | | | | | |
| **Secondo te cosa diranno gli altri se utilizzerai il pancreas artificiale?** | | | | | |
|  | In completo disaccordo | In disaccordo | Neutrale | D’accordo | Completamente d’accordo |
| 19) Le persone che sono per me importanti (famiglia e amici), saranno d’accordo che mi serva utilizzare il pancreas artificiale | 1 | 2 | 3 | 4 | 5 |
| 20) Gli insegnanti mi coinvolgeranno nella condivisione della novità con la classe | 1 | 2 | 3 | 4 | 5 |
| 21) In classe sarà sicuramente un problema perché i compagni non amano le novità | 1 | 2 | 3 | 4 | 5 |
| 22) I compagni non diranno nulla, saranno solamente interessati in un primo momento | 1 | 2 | 3 | 4 | 5 |
| 23) I compagni potrebbero porre domande con insistenza | 1 | 2 | 3 | 4 | 5 |
| 24) Altre persone potrebbero guardarmi con insistenza | 1 | 2 | 3 | 4 | 5 |
| 25) Mi renderà un esempio verso le altre persone con diabete | 1 | 2 | 3 | 4 | 5 |
| **Pensi che il pancreas artificiale sia semplice da utilizzare?** | | | | | |
|  |  |  |  |  |  |
| 26) Penso sia semplice da utilizzare | 1 | 2 | 3 | 4 | 5 |
| 27) Penso non sarà facile imparare, ma con il tempo potrebbe essere più semplice | 1 | 2 | 3 | 4 | 5 |
| 28) Potrebbe migliorare la mia qualità di vita | 1 | 2 | 3 | 4 | 5 |
| 29) Potrebbe migliorare la qualità di vita della mia famiglia | 1 | 2 | 3 | 4 | 5 |
| 30) Potrebbe rendere più facile l’alimentazione | 1 | 2 | 3 | 4 | 5 |
| 31) Potrebbe rendere più facile l’esercizio fisico e lo sport | 1 | 2 | 3 | 4 | 5 |
| 32) Potrebbe rendermi più autonomo e meno dipendente dalle altre persone | 1 | 2 | 3 | 4 | 5 |
| 33) Potrebbe rendere più facile la gestione del diabete a scuola o al lavoro | 1 | 2 | 3 | 4 | 5 |
| 34) Potrebbe rendere più facile la gestione del diabete durante i giorni di malattia | 1 | 2 | 3 | 4 | 5 |
| 35) Potrebbe farmi dormire meglio | 1 | 2 | 3 | 4 | 5 |
| 36) Permetterà ai miei genitori di dormire di più | 1 | 2 | 3 | 4 | 5 |
| 37) Penso che sarò in grado di utilizzare bene il pancreas artificiale | 1 | 2 | 3 | 4 | 5 |
| 38) Penso mi servirà un corso prima di utilizzare il pancreas artificiale | 1 | 2 | 3 | 4 | 5 |
| **Sei fiducioso sul funzionamento del pancreas artificiale?** | | | | | |
|  | In completo disaccordo | In disaccordo | Neutrale | D’accordo | Completamente d’accordo |
| 39) Ho fiducia delle misurazioni del glucosio che effettua il pancreas artificiale | 1 | 2 | 3 | 4 | 5 |
| 40) Ho fiducia che il pancreas artificiale somministrerà la quantità corretta di insulina | 1 | 2 | 3 | 4 | 5 |
| 41) Gli allarmi aiuteranno la mia fiducia nel pancreas artificiale | 1 | 2 | 3 | 4 | 5 |
| 42) Gli allarmi mi potrebbero disturbare nell’attività quotidiana | 1 | 2 | 3 | 4 | 5 |
| 43) Gli allarmi mi potrebbero disturbare soprattutto la notte | 1 | 2 | 3 | 4 | 5 |
